# Hippo pathway in non-small cell lung cancer: mechanisms, potential targets, and biomarkers

**DOI:** 10.1038/s41417-024-00761-z

**Published:** 2024-03-18

**Authors:** Hongge Liang, Yan Xu, Jing Zhao, Minjiang Chen, Mengzhao Wang

**Affiliations:** Department of Respiratory and Critical Care Medicine, Peking Union Medical College Hospital, Chinese Academy of Medical Sciences and Peking Union Medical College, Beijing, China

**Keywords:** Non-small-cell lung cancer, Cancer therapeutic resistance

## Abstract

Lung cancer is the primary contributor to cancer-related deaths globally, and non-small cell lung cancer (NSCLC) constitutes around 85% of all lung cancer cases. Recently, the emergence of targeted therapy and immunotherapy revolutionized the treatment of NSCLC and greatly improved patients’ survival. However, drug resistance is inevitable, and extensive research has demonstrated that the Hippo pathway plays a crucial role in the development of drug resistance in NSCLC. The Hippo pathway is a highly conserved signaling pathway that is essential for various biological processes, including organ development, maintenance of epithelial balance, tissue regeneration, wound healing, and immune regulation. This pathway exerts its effects through two key transcription factors, namely Yes-associated protein (YAP) and transcriptional co-activator PDZ-binding motif (TAZ). They regulate gene expression by interacting with the transcriptional-enhanced associate domain (TEAD) family. In recent years, this pathway has been extensively studied in NSCLC. The review summarizes a comprehensive overview of the involvement of this pathway in NSCLC, and discusses the mechanisms of drug resistance, potential targets, and biomarkers associated with this pathway in NSCLC.

## Introduction

Among malignant tumors worldwide, lung cancer has the highest mortality rate and the second-highest annual incidence, and non-small cell lung cancer (NSCLC) accounts for approximately 85% of all lung cancer cases [1]. Unfortunately, the majority of NSCLC patients are diagnosed at advanced stages, resulting in a poor prognosis for these individuals [2, 3]. Recently, several carcinogenic pathways have been identified in NSCLC, including mutations in the epidermal growth factor receptor (EGFR), fusion of the anaplastic lymphoma kinase (ALK), mutations in the Kirsten rat sarcoma viral oncogene homolog (KRAS), rearrangement of the ROS proto-oncogene 1 (ROS1), mutations in the B-Raf proto-oncogene (BRAF), fusion of the neurotrophin receptor kinase (NTRK), exon 14 skipping mutation in the c-mesenchymal-epithelial transition factor (c-MET), rearrangement of the rearranged during transfection proto-oncogene (RET), and mutations in the human epidermal growth factor receptor 2 (ERBB2/HER2). These pathways play a significant role in the development and progression of NSCLC. Currently, drugs developed for targeting these oncogenic pathways have been approved by the National Comprehensive Cancer Network (NCCN) guidelines for treating advanced NSCLC with corresponding genetic alterations [4, 5]. Furthermore, several phase III clinical trials have demonstrated significant and durable clinical benefits in advanced NSCLC patients through the application of immune checkpoint inhibitors (ICIs) that target programmed cell death-ligand 1 (PD-L1), programmed cell death 1 (PD-1), and cytotoxic T-lymphocyte-associated protein 4 (CTLA-4). NCCN guidelines have approved atezolizumab (ICI targeting PD-L1), nivolumab (ICI targeting PD-1), pembrolizumab (ICI targeting PD-1), and ipilimumab (ICI targeting CTLA-4) for treating NSCLC as they significantly prolong survival in some patients [5]. However, although targeted therapy and immunotherapy greatly improved the prognosis in some patients with NSCLC, after an initial positive response to treatment, it is inevitable that patients will eventually develop acquired drug resistance over time [6]. Previous studies have shown that the major effector factors of the Hippo pathway, namely Yes-associated protein (YAP) and transcriptional co-activator PDZ-binding motif (TAZ), play a key role in mediating drug resistance in NSCLC [7].

**Fig. 1:**
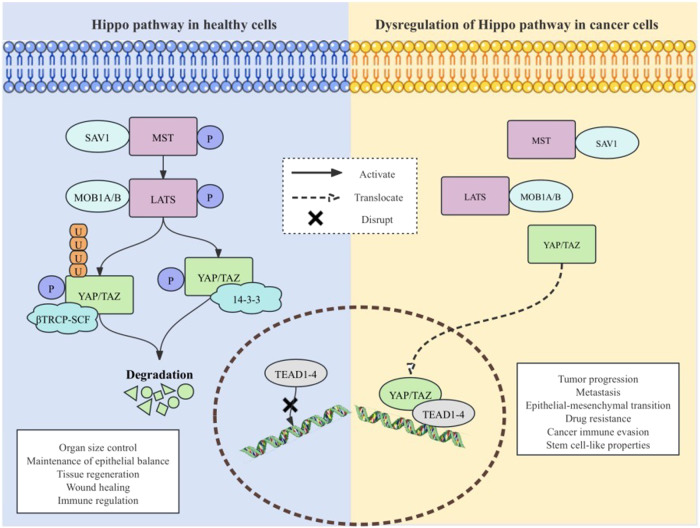
Hippo pathway in cells. There are no designated ligands or receptors for the Hippo pathway. The Hippo pathway can be activated by various signals, including cell-cell contact, extracellular matrix (ECM), cell polarity, cell density, and mechanical signals. When the Hippo pathway is activated in healthy cells, MST1/2 and their adaptor protein SAV1 phosphorylate LATS1/2. Once phosphorylated, LATS1/2 and their adaptor MOB1A/B phosphorylate YAP and TAZ. These phosphorylation events lead to the sequestration of YAP and TAZ in the cytoplasm and promote their subsequent degradation. This sequestration and degradation can occur through the binding of YAP and TAZ to 14-3-3 proteins or the action of the ubiquitin ligase βTRCP-SCF complex, regulating organ size control, maintaining epithelial balance, modulating tissue regeneration, enhancing wound healing, and contributing to immune regulation in healthy tissues. On the other hand, when the Hippo pathway is dysregulated in cancer cells, YAP and TAZ undergo translocation into the nucleus, binding to TEAD1-4 proteins. TEAD1-4 brings YAP and TAZ to the appropriate DNA elements to upregulate proliferative molecules. Dysregulation of the Hippo pathway plays a key role in tumor progression, drug resistance, cancer immune evasion, metastasis, epithelial–mesenchymal transition, and stem cell-like properties. MST mammalian STE20-like protein kinase, LATS large tumor suppressor kinase, SAV1 Salvador homolog-1, MOB1A/B MOB Kinase Activator 1A and 1B, YAP Yes-associated protein, TAZ transcriptional co-activator PDZ-binding motif, TEAD transcriptional-enhanced associate domain, P phosphorylation.

The Hippo pathway, initially identified in Drosophila and conserved throughout mammalian evolution, consists of key components in mammals including MST1 and MST2 (STe20-like kinases 1 and 2), SAV1 (Salvador homolog-1) as their binding partner, LATS1 and LATS2 (large tumor suppressor kinase 1 and 2) along with their binding partners MOB1A/B (MOB Kinase Activator 1A and 1B), YAP, TAZ, and transcriptional-enhanced associate domain (TEAD) [8–10]. There are no designated ligands or receptors for the Hippo pathway [11]. The Hippo pathway can be activated by various signals, both from the extracellular and intracellular environment. These signals include cell-cell contact, the extracellular matrix (ECM), cell polarity, cell density, mechanical signals, intracellular tension, soluble factors, stress signals, cellular energy status, mitogens, changes in cellular metabolism, tyrosine kinase receptors, and ligands of G-protein-coupled receptors (GPCR), among others [11–15]. When the Hippo pathway is activated, MST1/2 and their adaptor protein SAV1 phosphorylate LATS1/2 [8, 16]. Once phosphorylated, LATS1/2 and their adaptor MOB1A/B phosphorylate YAP and TAZ. These phosphorylation events lead to the sequestration of YAP and TAZ in the cytoplasm and their subsequent degradation. This sequestration and degradation can occur through the binding of YAP and TAZ to 14-3-3 proteins or the action of the ubiquitin ligase βTRCP-SCF complex [11]. On the other hand, when the Hippo pathway is deactivated, YAP and TAZ undergo translocation into the nucleus, binding to TEAD1-4 proteins. TEAD1-4 brings YAP and TAZ to the appropriate DNA elements to upregulate proliferative molecules [8, 10, 11, 17]. The Hippo pathway regulates proliferation, cellular overgrowth, contact inhibition, stem cell function, tissue homeostasis, wound healing, apoptosis, differentiation, immune response, and regeneration in healthy tissues [10, 18–24]. In addition, the Hippo pathway plays a pivotal role in the development and progression of tumor [25, 26] (Fig. 1). The dysregulation of the Hippo signaling pathway results in the activation of YAP, which promotes the progression of various types of human cancers, including lung cancer, mesothelioma, breast cancer, liver cancer, neural tumors, gastric cancer, ovarian cancer, urogenital cancer, and skin cancer [27–36]. Previous studies have demonstrated the significant involvement of the Hippo pathway in drug resistance, cancer immune evasion, metastasis, and epithelial–mesenchymal transition (EMT) in NSCLC [37–42]. Furthermore, reduced expression of LATS1 and increased expression of YAP are correlated with unfavorable prognosis in NSCLC [43, 44].

**Fig. 2:**
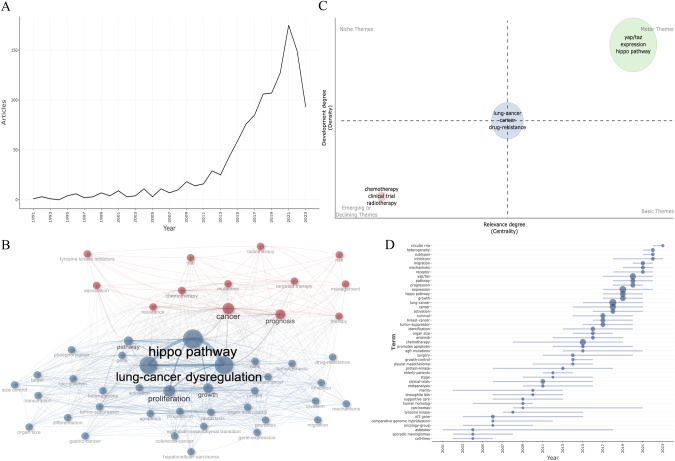
Bibliometric analysis. **A** Annual Scientific Production indicated that the quantity of articles measuring the role of the Hippo pathway in lung cancer has steadily risen since 1991, with a significant surge in research from 2013. **B** Co-occurrence network revealed that current research predominantly focuses on the effects of the dysfunctional Hippo pathway on tumor proliferation, treatment resistance, and prognosis. Other areas of interest encompass the influence of the Hippo pathway on tumor growth, heterogeneity, apoptosis, metastasis and differentiation, etc. **C** Thematic map suggested that the current research focuses on the expression level of YAP and TAZ in the Hippo pathway. Furthermore, there is extensive research potential in exploring the role of the Hippo pathway in lung cancer resistance to radiotherapy and chemotherapy. **D** Trend topics indicated that over the last five years, research hotspots have primarily concentrated on the interaction between the Hippo pathway and related circular rRNA, the impact of the Hippo pathway on tumor heterogeneity, the classification of different tumor subtypes, inhibitors of the Hippo pathway, the effects of the Hippo pathway on tumor migration, and the expression levels of key components of the Hippo pathway.

**Fig. 3:**
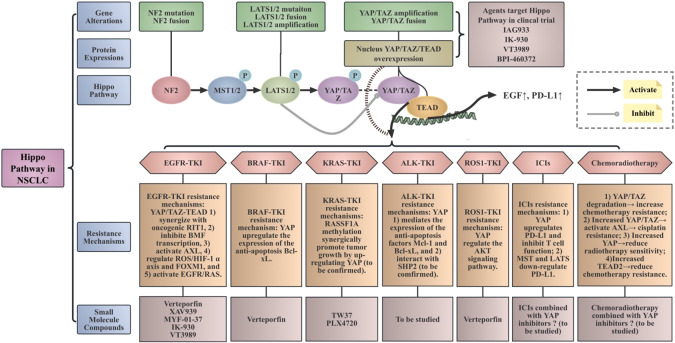
Hippo Pathway in NSCLC. The NF2 gene functions as a tumor suppressor and serves as a key negative regulator of the Hippo pathway. When the Hippo pathway is activated, MST1/2 and their adaptor protein SAV1 phosphorylate LATS1/2. Once phosphorylated, LATS1/2 and their adaptor MOB1A/B phosphorylate YAP and TAZ. These phosphorylation events lead to the sequestration of YAP and TAZ in the cytoplasm and their subsequent degradation. When the Hippo pathway is disrupted, YAP/TAZ enters the nucleus and binds to TEAD, leading to the expression of target genes. Dysregulation of the Hippo pathway in NSCLC mainly involves: (1) Gene alterations: including NF2 mutation, NF2 fusion, LATS1/2 mutation, LATS1/2 fusion, LATS1/2 amplification, YAP/TAZ fusion, YAP/TAZ amplification; (2) Protein expressions: nucleus YAP/TAZ/TEAD overexpression. Dysregulation of the Hippo pathway is associated with resistance to various therapeutic strategies in NSCLC, such as targeted small-molecule inhibitors (EGFR-TKI, BRAF-TKI, KRAS-TKI, and ROS1-TKI), immune checkpoint inhibitors, and traditional radiotherapy and chemotherapy. Apart from Hippo pathway dysfunction, it also has complex crosstalk with other pathways. Currently, a range of small-molecule compounds related to the Hippo pathway are being studied, with some already in clinical trial phases. NSCLC non-small cell lung cancer, NF2 neurofibromin 2, MST mammalian STE20-like protein kinase, LATS large tumor suppressor kinase, YAP Yes-associated protein, TAZ transcriptional co-activator PDZ-binding motif, TEAD transcriptional-enhanced associate domain, P phosphorylation, EGFR epidermal growth factor receptor, BRAF B-Raf proto-oncogene, KRAS Kirsten rat sarcoma viral oncogene homolog, ALK anaplastic lymphoma kinase, ROS1 ROS proto-oncogene 1, TKI tyrosine kinase inhibitor, ICIs immune checkpoint inhibitors, ROS reactive oxygen species, HIF hypoxia-inducible factor, Bcl-xL B-cell lymphoma-XL, RASSF1A ras association domain family 1A, Mcl-1 myeloid cell leukemia-1, SHP2 Src homologous phosphotyrosine phosphatase 2, PD-L1 programmed cell death-Ligand 1.

In order to gain a comprehensive understanding of the impact of the Hippo pathway on lung cancer, we conducted a search for articles related to the Hippo pathway and lung cancer on the Web of Science Core Collection (Supplementary Material S1 provides the keywords used in the search). A total of 1211 literature records were retrieved. Subsequently, a metrology analysis was performed on this collection of literature. The analysis revealed several key findings. The number of literature articles studying the interaction between lung cancer and the Hippo pathway has been increasing over the years (Fig. 2A). The existing studies primarily focused on the effects of Hippo pathway dysfunction on tumor proliferation, treatment resistance, and prognosis (Fig. 2B). In terms of research topics, the expression levels of YAP/TAZ in the Hippo pathway emerged as a current research hotspot. In addition, the investigation of drug resistance in lung cancer exhibited considerable research prospects (Fig. 2C). According to the research trends, the latest focus is on exploring the interaction mechanism between the Hippo pathway and related circular rRNA, followed by the impact of the Hippo pathway on tumor heterogeneity and the classification of different tumor subtypes (Fig. 2D). This article examines the pertinent studies on the Hippo pathway and emphasizes its significance in mechanisms of drug resistance, potential targets, and biomarkers in NSCLC (Fig. 3).

## Dysregulation of the Hippo pathway in NSCLC

Dysregulation of the Hippo pathway is of great significance in the initiation, progression, and metastasis of tumors, rendering it a promising therapeutic target. Accumulating preclinical and clinical findings indicate that the deficiency of upstream regulatory factors in the Hippo pathway or the overactivation of YAP /TAZ plays a role in tumor growth and metastasis [45] [11, 25]. Although the Hippo pathway is dysregulated in many cancer types, genetic alterations affecting this pathway are relatively rare and present in less than 10% of patients [26]. Oncogenic alterations of the Hippo pathway in NSCLC include neurofibromin 2 (NF2) mutation, NF2 fusion, LATS1/2 mutation, LATS1 fusion, YAP1/TAZ fusion, and YAP1/TAZ gene amplification [46–50]. Moreover, changes in the expression levels of key molecules in the Hippo pathway have been identified in NSCLC, potentially impacting the prognosis of NSCLC patients.

### NF2

The NF2 gene, which encodes the Merlin protein, functions as a tumor suppressor and serves as a key negative regulator of the Hippo pathway. Its function contributes to the phosphorylation of MST and LATS, resulting in the cytoplasmic localization and degradation of YAP and TAZ [11, 51]. In addition, NF2 can regulate the palmitoylation of TEAD by controlling the expression of FASN, thereby inhibiting YAP/TAZ-induced transcription in response to cell contact [52]. Thus, loss-of-function mutations of NF2 can activate YAP/TAZ-TEAD, leading to the onset and progression of tumors [53]. Previous studies have shown that numerous acquired somatic mutations occur in NF2, particularly in meningioma, mesothelioma, and peripheral neurinoma. These mutations are also found in other types of cancer, such as breast cancer, hepatocellular carcinoma, intrahepatic cholangiocarcinoma, and renal cell carcinoma [54, 55]. Patients with malignant mesothelioma harboring NF2 mutations respond to TEAD inhibitor [56]; however, the NF2 mutation rate in lung squamous cell carcinoma is only 2.2% [57]. In addition, NF2 fusion inhibiting NF2 function, including NF2-OSBP2 and NF2-MORC2-fusion, have been found in lung cancer [49]. Further research is required to determine the effectiveness of drugs that target the Hippo pathway in NSCLC patients with NF2 gene alterations.

### MST/LATS

The upstream components of the Hippo pathway, MST1/2 and LATS1/2, function as tumor suppressors by promoting the binding of YAP/TAZ and 14-3-3 through phosphorylation, leading to their cytoplasmic localization and degradation [58]. Genetic alterations or functional deletions of these molecules can promote the nuclear translocation of YAP and TAZ. Nuclear YAP and TAZ bind to the TEAD transcription factor family and trigger the expression of target genes, potentially contributing to the occurrence and development of tumors [59, 60]. Moreover, the copy number loss of LATS1/LATS2 has been documented in numerous cancer types, which was shown to increase cell proliferation in vitro [61, 62]. Furthermore, targeted therapy for the Hippo pathway is effective in tumors with LATS dysfunction. For example, the TEAD palmitoylation inhibitor IK-930 was effective in the CDX model of MSTO-211H mesothelioma with LATS deficiency [63]. In addition, gene fusions involving LATS1 and LATS2 have been found in lung cancer [49], and low expression level of LATS1 was associated with poor prognosis in patients with NSCLC [43]. Further studies are needed to investigate the effect of genetic changes in MST/LATS on the therapeutic efficacy of targeted therapy for Hippo pathway in NSCLC.

### YAP/TAZ

YAP and TAZ serve as co-factors of several transcription factors. When the Hippo pathway is inactivated, YAP and TAZ translocate to the nucleus, where they form a complex with TEAD. They contribute to the occurrence and progression of various tumors, such as lung cancer, breast cancer, liver cancer, colon cancer, and ovarian cancer [24, 27, 45, 54, 64, 65], by promoting cell survival, abnormal growth, and stem cell-like properties [25].

Activating mutations in YAP and TAZ are extremely uncommon in human cancer [54, 61, 66], and individual activation mutations of YAP and TAZ are insufficient to induce tumorigenesis, which may be related to the negative feedback loop associated with YAP and TAZ [67]. Nonetheless, the interaction of the Hippo pathway with the dysfunction of other pathways can trigger tumorigenesis [68]. For instance, increased YAP expression in type II alveolar epithelial cells only leads to hyperplasia in mouse lungs. However, ectopic YAP expression accelerates the progression of de novo lung cancer in the Kras^G12D^ mouse model [68]. In addition, YAP/TAZ fusions are the major causes of Hippo pathway dysregulation. These fusion events preserve the TEAD binding domain, and have the potential to promote the transcription of TEAD target genes, thereby enhancing tumor progression [69]. Fusions of YAP or TAZ with other genes, including TAZ-CAMTA1, YAP-MAMLD1, YAP-MAML2, YAP-FAM118B, YAP-KMT2A, YAP-TFE3, and YAP-NUTM1, have been reported in various rare tumors such as epithelioid hemangioendothelioma, glioma, supratentorial ependymoma, etc. Similarly, fusion events affecting key components of the Hippo pathway have been found in patients with lung cancer. For instance, fusions of oncogenic proteins TAZ, YAP1, and HIPK2 preserve their tumor-promoting function, while fusions of tumor suppressors, such as NF2, LATS1, FAT1, PTPN14, DCHS2, TAOK1, and TAOK3, results in loss of their function [49]. The increased expression levels of YAP and TAZ are usually attributed to gene amplification [61] or decreased degradation through autophagosomes [70] and ubiquitin–proteasome degradation systems [71, 72]. Wang et al. [26] reported a combined amplification frequency of 0-19% for YAP and TAZ in 33 cancers, with lung squamous cell carcinoma ranking second. Besides, there is more YAP nucleus enrichment in NSCLC than in healthy tissues [73], which has a crucial function in maintaining NSCLC stemness [50]. Moreover, the overexpression levels of YAP and TAZ are associated with poor survival in patients with NSCLC [47, 48].

### TEAD

As the primary partner in YAP/TAZ-mediated oncogenic transcription [74], the TEAD transcription factor family serves as the ultimate nuclear effector of the Hippo pathway and is widely expressed in human tissues [75, 76]. TEAD alone has almost no transcriptional activity and relies on transcriptional co-activators to trigger the expression of target genes [77]. Among them, the most established co-factors for activating TEAD are YAP and its paralog TAZ. The N-terminus of YAP/TAZ interacts with the C-terminus of TEAD, forming the YAP/TAZ-TEAD complex, which forms the nuclear transcription module of the Hippo pathway [11]. In addition, the activity of TEAD depends on its nuclear/cytoplasmic translocation [78] and palmitoylation [52, 79]. TEAD activation is crucial for tumor development, as it plays a vital role in tumor EMT, metastasis, drug resistance, and stem cell self-renewal [80–84]. So far, there are no oncogenic mutations have been identified for TEAD. However, several studies indicated that the nuclear preservation and activation of YAP and TAZ may rely on their interaction with TEAD [85, 86]. Previous studies have demonstrated that elevated TEAD expression and nuclear accumulation are associated with poorer overall survival in various cancer types, including breast cancer [87], rectal cancer [88], pancreatic ductal adenocarcinoma [89], and head and neck cancer [90]. These findings underscore the potential significance of TEAD in cancer prognosis. However, the effect of TEAD nuclear expression levels on the prognosis of NSCLC and its potential clinical application as a biomarker in NSCLC still need more studies.

### Role of the Hippo pathway in resistance to targeted therapy in NSCLC

Studies have reported that disruptions in the Hippo pathway are correlated with unfavorable clinical outcomes in patients with NSCLC [44, 73]. Besides its role in tumorigenesis, the Hippo pathway plays a pivotal role in drug resistance of NSCLC via crosstalk with other well-known tumor-promoting factors such as EGFR, ALK, BRAF, KRAS, and ROS1 [91–98]. Several studies have shown that in NSCLC cells with gene alterations of KRAS G12C, BRAF V600E, EGFR, ALK, or ROS1, dysregulation of the Hippo pathway decreases the initial response to various tyrosine kinase inhibitors, which is associated with resistance of targeted therapy and tumor recurrence. YAP inhibition restored the sensitivity of NSCLC cells to targeted treatment in these studies [91, 93, 94, 97, 99, 100].

### EGFR mutation

EGFR, a receptor belonging to the ErbB family, is encoded by EGFR exon 18–24. Mutations that activate the kinase domain of EGFR primarily occur at exon 18-21, leading to the activation of downstream signaling pathways such as Ras/Raf/MEK/ERK, Janus kinase (JAK)/STAT, and phosphatidylinositol 3-kinase (PI3K)/Akt/mTOR. These mutations are strongly associated with human NSCLC [101–104]. The EGFR pathway has been well studied as an oncogenic pathway in NSCLC [105–109]. Patients with EGFR-sensitive mutations in NSCLC can experience therapeutic benefits from the use of EGFR-tyrosine kinase inhibitors (TKIs). These include 1st-generation EGFR-TKIs (gefitinib and erlotinib), 2nd-generation EGFR-TKIs (dacomitinib and afatinib), and 3rd-generation EGFR-TKI (osimertinib) [98, 110–117]. Nevertheless, it is inevitable for patients undergoing EGFR-TKI treatment to eventually develop drug resistance, and it is essential to elucidate the mechanism of acquired resistance. Previous studies reported that EGFR-TKI resistance is associated with YAP overexpression or YAP amplification, and silencing YAP by siRNA can reverse EGFR-TKI resistance in NSCLC cells [40, 118]. Similarly, another study discovered that the increased expression of YAP in EGFR-mutated NSCLC cells led to resistance to EGFR-TKI (erlotinib), whereas the knockdown of YAP increased the sensitivity of NSCLC cells to erlotinib [99]. Meanwhile, clinical studies found that YAP overexpression was associated with NSCLC progression and poor clinical outcomes. Chaib et al. reported that increased expression of STAT3 or YAP1 was associated with poor progression-free survival (PFS) in NSCLC patients harboring EGFR mutation who received first-line EGFR-TKIs, suggesting that both Src-YAP axis and STAT3 can lead to a reduced response to EGFR-TKIs in lung cancer [100].

The interaction between the Hippo pathway and the EGFR signaling pathway in lung adenocarcinoma (LUAD) is intricate. Previous research has demonstrated that inhibiting the Hippo pathway can activate the EGFR signaling pathway, resulting in resistance to EGFR-TKIs. For example, Vichas et al. found that inactivation of Hippo signaling and overexpression of YAP may enhance the oncogenic effects of RIT1. They also found that RIT1 M90I mutation may activate the EGFR/RAS pathway and lead to PC9 cell (EGFR-mutant LUAD cell) resistance to erlotinib or osimertinib [119]. Kurppa et al. showed that YAP activation downregulated the transcription of pro-apoptotic protein BMF by binding to EMT-related transcription factor SLUG, and then induced NSCLC cell dormancy to escape the cytotoxic effects of EGFR/MEK inhibitors. EGFR/MEK inhibitors combined with the covalent TEAD inhibitor MYF-01-37 promoted the apoptosis of dormant tumor cells [120]. In lung cancer cells harboring EGFR-sensitive mutations, YAP activation leads to the upregulation and activation of the tyrosine kinase receptor AXL. AXL activation is associated with drug resistance in lung cancer cells [93, 121]. Moreover, YAP can contribute to resistance to EGFR-TKIs in NSCLC by regulating ROS/HIF-1α axis and FOXM1 [122, 123]. Furthermore, the YAP/TAZ-TEAD complex can enhance the transcription of genes associated with the EGFR signaling pathway. Several researchers have reported that YAP activation can upregulate the expression of downstream ligands of the EGFR family receptors, including neuregulin 1 (NRG-1), amphiregulin (AREG), and ERBB3/4, through the binding of YAP to TEAD and the formation of an autocrine ring. A positive feedback loop is generated to stabilize Hippo inhibition and enhance MAPK activity, thereby inducing tumor progression and drug resistance [99, 118, 124]. Similarly, blocking the EGFR signaling pathway can lead to the degradation of YAP. Previous studies indicated that inhibiting ERK1/2 reduces the expression level of YAP and subsequently decreases the expression of downstream genes in the Hippo pathway. Inhibition of YAP activity hinders the migration and invasion of NSCLC cells. Meanwhile, forced expression of the ERK2 gene can rescue YAP protein levels [92]. In addition to YAP, TEADs play a crucial role in resistance to EGFR-TKIs. It has been suggested that TEADs are mediators of the EGFR/RAS/RAF/MAPK pathway and play a crucial role in tumor progression and drug resistance in NSCLC with EGFR, KRAS, or BRAF mutation [125]. Therefore, targeting YAP/TEAD is a potential therapeutic approach to overcome acquired resistance to EGFR-TKIs [40, 91, 118, 126].

### BRAF V600E mutation

BRAF is a protein kinase that plays a crucial role in cellular signaling. It is a serine–threonine kinase protein with a molecular weight of 95 kDa. BRAF is located on the long arm of chromosome 7 (7q) [127], and is an important component of the MAP/ERK signaling pathway. Activation of BRAF, a downstream molecule of the receptor tyrosine kinase (RTK), involves RAS-dependent phosphorylation at Ser601 and Thr598 [128]. Upon activation, BRAF can phosphorylate MEK 1/2, which will then phosphorylate ERK1/2 and regulate cell proliferation, division, and apoptosis [127]. The BRAF V600E mutation is detected in ~1–2% of patients diagnosed with lung adenocarcinoma, and targeted therapies against BRAF have shown promising results in treating these patients [129, 130]. Currently, NCCN guidelines have approved dabrafenib, trametinib, and vemurafenib for treating advanced NSCLC with BRAF V600E mutation [130–132]. Previous studies have demonstrated that the increased expression of YAP1 or its analog TAZ significantly diminishes the sensitivity of the lung adenocarcinoma cell line carrying the BRAF V600E mutation (HCC364) to vemurafenib and trametinib. Further mechanistic studies have revealed that YAP can promote the resistance of lung cancer cells to RAF and MEK inhibitors by upregulating the expression of the anti-apoptotic protein BCL-xL. The overexpression of BCL-xL counteracted the impact of YAP1 silencing on the response of HCC364 cells to RAF and MEK inhibitors. Meanwhile, inhibition of YAP or BCL-xL (TW37, ABT-263) synergistically increased the cytotoxic effect of RAF and MEK inhibitors on NSCLC cells [91].

### KRAS G12C mutation

KRAS, a membrane protein with intrinsic guanosine triphosphatase (GTPase) activity, is one of the most common oncogenes in humans. Approximately one in seven human cancers and around 25% of human NSCLC cases are characterized by KRAS mutations [133]. The most common mutation site is codon 12 [134]. KRAS G12C mutation can abnormally increase the concentration of GTP-bound KRAS, which overactivates downstream carcinogenic pathways and promotes cell growth [135]. The incidence of KRAS is 14% in lung adenocarcinoma and 0.5–4% in lung squamous carcinoma [136]. Currently, NCCN guidelines have approved Sotorasib [137] and Adagrasib [138] for treating NSCLC patients with KRAS G12C mutations.

Earlier studies have indicated that patients with NSCLC carrying KRAS mutations exhibit elevated levels of YAP expression, and YAP1 silencing can enhance response to trametinib in human lung adenocarcinoma cells (MOR/CPR) encoding KRAS G12C [91]. These findings suggest that increased YAP1 expression may be related to trametinib resistance, which needs further confirmation. Besides, the tumor suppressor RASSF1A can inhibit YAP activity through the GEF-H1/RhoB pathway and promote YAP degradation in the cytoplasm [139]. In NSCLC, the mRNA expression level of RASSF1A was found to be significantly reduced in stage I tumors when compared to normal tissues and was negatively correlated with gene methylation [140]. Moreover, simultaneous RASSF1A methylation and KRAS mutation shortened survival in patients with NSCLC [141], suggesting that RASSF1A methylation may synergically promote the growth of lung cancer with KRAS mutation by inhibiting the Hippo pathway, which needs further confirmation.

### ALK rearrangement

ALK is another potent oncogene in NSCLC. ALK rearrangement is a distinct molecular subtype in NSCLC. It causes an aberrant expression of the tyrosine kinase-containing portion of the ALK gene, resulting in its continuous activation. This fusion event is observed in ~4–6% of individuals with lung adenocarcinoma [142]. Patients with NSCLC who have ALK gene fusion typically are sensitive to ALK-TKIs. Currently, the U.S. Food and Drug Administration (FDA) has approved five ALK-TKIs for the treatment of advanced NSCLC with ALK rearrangement. These approved ALK-TKIs include crizotinib, brigatinib, alectinib, lorlatinib, and ceritinib [143–148]. Although ALK-TKIs have shown significant benefits in prolonging the overall survival of patients with NSCLC harboring ALK rearrangement, they inevitably lose their efficacy due to drug resistance. To address this issue, ongoing research is focused on understanding the mechanisms of resistance and developing strategies to overcome or prevent it. As previously reported in the literature, resistance to ALK-TKIs can occur through both ALK-dependent mechanisms, such as ALK secondary mutation and ALK gene amplification, as well as ALK-independent mechanisms, including activation of alternative signaling pathways [149, 150]. The emergence of ALK-dependent resistance has led to the advancement of next-generation ALK-TKIs; however, ALK-independent resistance remains a challenge [151].

YAP amplification is an ALK-independent resistance mechanism of ALK-TKIs. Previous studies found that in patients with NSCLC harboring ALK rearrangement, high expression levels of YAP promoted resistance to ALK-TKIs, and the anti-tumor effect of ALK-TKIs was restored after YAP1 knockdown by siRNA in vivo and in vitro [97]. It has been reported that many genes involved in pathways enriched in cells resistant to crizotinib are related to YAP. Subsequent investigations demonstrated that inhibiting YAP1 genetically or pharmacologically suppressed tumor growth in ALK-TKI-resistant lung adenocarcinoma cells, EML4-ALK transgenic mice, and tumor xenograft models. In contrast, overexpressing YAP reduced the sensitivity of parental cells to ALK-TKIs. In addition, in NSCLC patients harboring ALK fusion, nuclear YAP is highly expressed in ALK-TKI-resistant samples, and high expression levels of YAP are related to weak response to ALK-TKIs [94]. Furthermore, it has been reported that after treating NSCLC cells carrying ALK rearrangement with alectinib in vivo and in vitro, the YAP1 can be activated, which mediates alectinib resistance by upregulating anti-apoptotic factors Mcl-1 and Bcl-xL [152]. In a separate study, it was discovered that blocking Src homologous phosphotyrosine phosphatase 2 (SHP2) in patient-derived cells (PDCs) resistant to ALK-TKIs reinstated the responsiveness of resistant cells to ALK inhibitors [153]. Interestingly, SHP2 has been found to interact with the YAP/TAZ, which plays a crucial role in the carcinogenic function of SHP2 [154]. Hence, targeting YAP represents a promising therapeutic approach for overcoming acquired resistance to ALK-TKIs.

### ROS1 rearrangement

ROS1, a receptor tyrosine kinase with constitutive kinase activity [155], is responsible for encoding both orphan ALK-related receptor tyrosine kinases and members of the insulin receptor family [156]. ROS1 rearrangement causes its components (including the entire tyrosine kinase domain) to fuse with one of the 12 different companion proteins. Common fusion partners include CD74, SLC34A2, CCDC6, and GOPC [157]. The resulting ROS1 fusion kinase is constitutionally active and drives cell transformation. ROS1 fusion is present in various human cancers, including NSCLC [158]. Rearrangement of the ROS1 gene is observed in ~1–2% of patients NSCLC [158–161], particularly in non-smokers, those with lung adenocarcinomas, and patients without alterations in the EGFR or ALK genes [159, 160, 162]. Due to the significant similarity between the kinase domains of ROS1 and ALK, the NCCN guidelines approved ALK inhibitors ceritinib [163], crizotinib [164], entrectinib [165], and lorlatinib [166] for treating NSCLC with ROS1 rearrangement.

Previous study has shown that treatment of lung cancer harboring ROS1 fusion with lorlatinib can activate YAP1 in vivo or in vitro. YAP1 protects cancer cells against lorlatinib by regulating the AKT signaling pathway. Conversely, YAP1 inhibition by siRNA or a YAP1 inhibitor, verteporfin, enhanced the response to lorlatinib in lung cancer cells (KTOR71). Furthermore, combination therapy with verteporfin and lorlatinib achieved a better anti-tumor effect in vivo compared to lorlatinib monotherapy, suggesting that YAP1 may mediate lorlatinib resistance of lung cancer cells (KTOR71), and simultaneous targeting of YAP1 and ROS1 may represent a more effective treatment strategy for patients with NSCLC harboring ROS1 rearrangement [167].

### Role of Hippo pathway in resistance to immunotherapy in NSCLC

Previous studies have demonstrated that essential elements of the Hippo pathway, including MST1/2, LATS1/2, YAP/TAZ, and TEAD, play important roles in innate and adaptive immunity against tumor cells [168–173]. Recently, ICIs have greatly improved the prognosis of some patients with NSCLC. When compared to chemotherapy, ICIs have shown a survival benefit in NSCLC. However, the objective response rate (ORR) in unselected NSCLC patients is only about 20% due to primary resistance to immunotherapy in some patients. In addition, patients who initially respond to immunotherapy may develop resistance over time, known as acquired resistance. Exploring the immunotherapy resistance mechanism is pivotal to enhancing the effectiveness of immunotherapy, expanding its application, and ameliorating ICI resistance [174]. Recent studies on oncogenic signaling pathways that regulate the expression of PD-L1 in tumors have opened up new possibilities for enhancing the effectiveness of immunotherapy and overcoming drug resistance in NSCLC. It was demonstrated that MST1/2 and LATS1/2 inhibit the expression level of PD-L1, while YAP/TAZ promotes PD-L1 expression in lung cancer cell lines [170, 175–177].

Immunohistochemical (IHC) staining of human NSCLC tissues revealed a significant correlation between nuclear YAP expression and PD-L1 expression. Furthermore, YAP inhibition using siRNA, drugs, or genome knockout techniques reduced both the mRNA and protein expression levels of PD-L1 in NSCLC cells [177]. Similarly, another research demonstrated that in NSCLC cells characterized by low levels of YAP and PD-L1 expression, the enforced expression of the YAP gene led to an elevation in PD-L1 protein levels [175]. YAP/TAZ-TEAD increases the activity of PD-L1 promoter, and TAZ-induced upregulation of PD-L1 can inhibit T cell function in human tumor cells. Hence, there is a possibility that YAP/TAZ could increase the expression of PD-L1 in NSCLC cells. Consequently, targeting YAP may enhance the effectiveness of anti-PD-1/PD-L1 monoclonal antibodies in treating advanced NSCLC patients [175–177]. Furthermore, additional studies have found that YAP regulates the expression level of PD-L1 in NSCLC resistant to EGFR-TKIs. Specifically, comparing to the parental PC9 cells (an NSCLC cell line with EGFR 19del mutation), it was observed that both YAP and PD-L1 were upregulated in the cells resistant to gefitinib. In addition, the knockdown of YAP in PC9 cells resistant to gefitinib led to a reduction in PD-L1 expression [176]. The findings presented in the study provide insights into the potential relationship between the EGFR signaling pathway, the Hippo pathway, and the expression of PD-L1 in NSCLC. These results suggest that the expression level of PD-L1 in NSCLC cells could be influenced, at least in part, by EGFR through the Hippo pathway. To explore the potential synergistic effects, it is necessary to investigate the efficacy of the combination of YAP inhibition and anti-PD-L1/PD-1 immune checkpoint inhibitors (ICIs) for NSCLC patients resistant to EGFR-TKIs.

Besides PD-L1, the Hippo pathway also affects other immune checkpoints. For instance, by analyzing the TCGA database, Kim et al. [178] discovered that inhibitory immune checkpoints, such as CTLA-4, PD-1, and PD-L2 were upregulated in the silence of the Hippo pathway (SOH) subgroup in glioblastoma. The authors also conducted IHC staining of YAP and PD-1 in glioblastoma tissue and found nuclear YAP staining showed a strong correlation with PD-1 staining. In addition to impacting on immune checkpoints, the Hippo pathway also affects the tumor immune microenvironment by regulating immune cell function and modulating the expression of cytokines and chemokines [179]. In various tumor types other than lung cancer, the Hippo pathway has been demonstrated to induce an immunosuppressive microenvironment by regulating M2 polarization of macrophage, Tregs differentiation and stability, B-cell differentiation and development, and the cytotoxicity of CD4+ and CD8 + T cells. This has been discussed in detail elsewhere [179, 180]. Nevertheless, due to the heterogeneity of various cancer types, further studies are needed to measure the effect of the Hippo pathway on the immune microenvironment of NSCLC.

### Role of Hippo pathway in resistance to chemoradiotherapy in NSCLC

Due to the limited beneficiaries of targeted therapy and immunotherapy, radiotherapy and chemotherapy still play a crucial role in the treatment of NSCLC patients. Extensive research has demonstrated that the Hippo signaling pathway plays a significant role in acquired chemoradiotherapy resistance in NSCLC. Activation of the YAP/TAZ pathway induces high expression of AXL in mesenchymal lung cancer, leading to doxorubicin resistance [181]. YAP1 expression is significantly elevated in NSCLC cells that are resistant to docetaxel, and the restoration of YAP1 expression can partially alleviate docetaxel resistance [182]. Curcumin has been shown to enhance the sensitivity of NSCLC cells to chemotherapy by facilitating the nucleocytoplasmic translocation and degradation of TAZ [183]. In addition, TEAD2 has the ability to counteract the increased sensitivity of NSCLC cells to cisplatin caused by miR-608 overexpression [184]. These studies collectively suggest the potential involvement of the Hippo pathway in NSCLC chemotherapy resistance. Furthermore, multiple investigations have established the role of the Hippo pathway in radiation resistance. Previous studies indicate that radiation therapy leads to the downregulation of YAP/TAZ, thereby inhibiting tumor proliferation [185]. On the other hand, the activation and nuclear translocation of YAP can reduce the sensitivity of tumors to radiotherapy [186, 187].

### Potential drugs for targeting the Hippo pathway

The YAP/TAZ-TEAD can activate the transcription of downstream genes in tumor cells, thereby maintaining tumor cell stemness and promoting the survival, proliferation, EMT, metastasis, and drug resistance of tumor cells [17, 19]. Currently, various drugs have been applied to target the Hippo pathway in different cancer types, which have been summarized in other reviews [67, 188]. Here, we focused on drugs targeting the Hippo pathway in NSCLC (Tables 1 and 2).

**Table 1:** Preclinical studies of drugs targeting the Hippo pathway.

Drug/small molecular	Mechanism	Model	Result	Reference
Digitoxin	YAP	Lung squamous cell lines (HTB-182, L78, CRL-5889); patient-derived xenograft (PDX)	Digitoxin effectively inhibited lung SCC progression by attenuating YAP phosphorylation and promoting YAP nuclear sequestration.	[190]
Verteporfin	YAP-TEAD	NSCLC cell lines (H1975)	Verteporfin, in combination with erlotinib, synergistically reduced migration, invasion, and tumor sphere formation abilities.	[99]
Verteporfin	YAP-TEAD	NSCLC cell lines (A549, H460)	Verteporfin inhibited the metastasis of A549 and H460 cells and the expression of EMT-related markers.	[189]
XAV939 MYF-01-37	YAP-TEAD	NSCLC cell lines (PC9, HCC827, HCC4006, HCC2279, H1975, H3122, EBC-1)	XAV939 or MYF-01-37 can deplete dormant cells by enhancing apoptosis induced by EGFR/MEK inhibition.	[119]
IK-930	TEAD palmitoylation	NSCLC cell lines (A549(KRAS G12S); H1975(EGFR))	IK-930 in combination with EGFR or MEK inhibitors can enhance cell apoptosis and anti-tumor activity.	[63]
VT3989	TEAD palmitoylation	NSCLC cell lines (H1975, HCC827)；CDX, PDX	VT3989, in combination with osimertinib, synergistically exerted anti-tumor effects.	[201]
VT104	TEAD palmitoylation	NSCLC CDX, PDX	VT104, in combination with osimertinib, induced a synergistic anti-tumor response.	[204]
MGH-CP1	TEAD palmitoylation	Various cells, including NSCLC cell lines (NCI-H226, NCI-H1299)	MGH-CP1 sensitivity is related to the YAP dependence.	[205]

*NSCLC* non-small cell lung cancer, *YAP* Yes-associated protein, *TEAD* transcriptional-enhanced associate domain, *CDX* cell line-derived xenograft, *PDX* patient-derived xenograft, *SCC* squamous cell carcinoma, *EMT* epithelial–mesenchymal transition, *EGFR* epidermal growth factor receptor, *MEK* mitogen-activated protein kinase kinases.

**Table 2:** Clinical studies of drugs targeting the Hippo pathway.

Agents	Mechanism	Clinical trial	Phase	Patients (including lung cancer)	Status
IAG933	YAP-TEAD	NCT04857372	I	Advanced malignant mesothelioma; solid tumors with NF2/LATS1/LATS2 gene alterations (truncated mutations or gene deletions) or functional YAP/TAZ fusions.	Recruiting
IK-930	TEAD palmitoylation	NCT05228015	I	Malignant mesothelioma with NF2 deletion; epithelioid hemangioendothelioma with TAZ-CAMTA1 or YAP1-TFE3 gene fusion; solid tumors with NF2 deletion or YAP1/TAZ fusion gene	Recruiting
BPI-460372BBB	TEAD palmitoylation	NCT05789602	I	Malignant mesothelioma; epithelioid hemangioendothelioma; solid tumors with NF2 deletion, YAP/TAZ fusion, LATS1/2 mutation, or other Hippo pathway dysregulation.	Recruiting
VT3989	TEAD palmitoylation	NCT04665206	I	Patients with mesothelioma with or without NF2 mutations who have progressed after standard treatment; refractory locally advanced or metastatic solid tumors with NF2 mutations.	Recruiting

*NF2* neurofibromin 2, *LATS* large tumor suppressor kinase, *YAP* Yes-associated protein, *TAZ* transcriptional co-activator PDZ-binding motif, *TEAD* transcriptional-enhanced associate domain.

### Targeting YAP/TAZ

YAP and TAZ are the core effectors of the Hippo pathway. They are negatively regulated by upstream components of the Hippo pathway, such as NF2, MST1/2, and LATS1/2 [17]. Currently, several in vitro and in vivo studies have confirmed the role of YAP and TAZ in promoting tumor cell proliferation, metastasis, and resistance to drugs [67]. In NSCLC, YAP1 is significantly upregulated and plays an oncogenic role compared to para-carcinoma normal tissues [189]. Huang et al. found that in lung squamous cell carcinoma cells, small-molecule digitoxin can downregulate YAP phosphorylation and activate YAP and nuclear sequestration. Activated YAP can lead to reactive oxygen species (ROS) accumulation by downregulating the antioxidant enzyme GPX2 and suppressing tumor progression. In patient-derived xenograft models, digitalis effectively inhibited lung squamous cell carcinoma progression and reduced YAP expression [190]. Therefore, targeting YAP has anti-tumor effects on human NSCLC in vitro and in vivo.

### Targeting YAP/TAZ-TEAD interactions

YAP and TAZ contain the N-terminal TEAD binding domain (TBD), which is a spherical structure that binds to TEAD and interacts at three different interfaces [191]. Verteporfin is a benzoporphyrin derivative, which is currently used for treating ocular vascular diseases [192, 193]. YAP1 expression is significantly upregulated in NSCLC. The binding of YAP-TEAD has been shown to enhance EMT, proliferation, invasion, and migration of lung cancer cells. Verteporfin exerts an anti-tumor effect on NSCLC by blocking AP-TEAD complex formation in vitro [189]. In human NSCLC cells, it was observed that the overexpression of YAP led to the development of resistance to erlotinib, a targeted therapy drug. Conversely, the sensitivity of H1975 cells (an NSCLC cell line with EGFR L858R/T790M mutation) to erlotinib was enhanced by inhibiting YAP through the use of small interfering RNA (siRNA) or verteporfin. In addition, the combination of verteporfin and erlotinib can synergically inhibit H1975 cell migration, invasion, and tumor globular formation [99]. YAP-TEAD inhibited the pro-apoptotic factor BMF through the EMT transcription factor SLUG, thereby preventing apoptosis [120]. In NSCLC with EGFR mutations, inhibition of EGFR/MEK led to dormant cell formation that exhibited increased YAP-TEAD activity. In addition, YAP-TEAD inhibitors, XAV939 and MYF-01-37, enhanced EGFR/MEK inhibitor-induced apoptosis, playing a synergistic role in tumor [120].

### Targeting TEAD palmitoylation

In tumors induced by Hippo pathway dysregulation, YAP and TAZ do not possess an intrinsic DNA binding domain and need to bind to TEAD to activate downstream oncogenes [74]. Therefore, TEAD is also an important drug target. TEAD is an evolutionarily conserved transcription factor that can undergo auto-palmitoylation at a conserved cysteine residue site. Post-translational modification of this protein is crucial for effective transcription, protein stability, and YAP/TAZ-TEAD interaction [52, 79, 194, 195]. The palmitate component of TEAD is deeply embedded within a hydrophobic pocket, and this unique biochemical and structural characteristic makes small molecules ideal inhibitors of TEAD. Therefore, various TEAD inhibitors have been developed for targeting the conserved palmitoylation sites and inhibiting the transcription of downstream target genes [196–200]. The potential anti-cancer role of TEAD palmitoylation has been demonstrated in NF2-deficient malignant mesothelioma [63, 199, 201].

It has been reported that VT3989 combined with osimertinib has strong synergistic anti-tumor effects against several EGFR-mutated NSCLC cell lines. In addition, VT3989 and osimertinib synergistically exerted anti-tumor effects in xenograft models of NCI-H1975 and HCC827 cell lines. Furthermore, when compared to the use of osimertinib alone, the combination of VT3989 and osimertinib has shown enhanced efficacy and delayed tumor regeneration. Moreover, in comparison to osimertinib monotherapy, the combination of VT3989 and osimertinib enhanced the effectiveness of osimertinib and delayed tumor regeneration [201]. A recently published phase I trial showed that VT3989 was safe and well-tolerated, with durable anti-tumor activity [202, 203]. IK-930 is a specific small-molecule inhibitor that selectively targets the palmitoylation of TEAD. In tumors with EGFR or KRAS mutation, IK-930, in combination with EGFR or MEK inhibitors, enhanced cell apoptosis and anti-tumor activity in vivo. In NSCLC cell line (A549) with KRAS G12S mutation, the combination of mitogen-activated protein kinase (MEK) inhibitor trimetinib and IK-930 exerted a synergistic anti-tumor effect. Furthermore, in the xenograft model of the H1975 NSCLC cell line, the combination of IK-930, osimertinib, and trametinib led to the complete regression of the tumors. In addition, in the xenograft model of H1975 NSCLC cell line, the combination of osimertinib, trametinib, and IK-930 led to complete regression of the tumor [63]. In both NSCLC PDX and CDX models, the combination of osimertinib and VT104, a TEAD inhibitor, exhibited a synergistic anti-tumor response [204]. Furthermore, Sun et al. reported that the TEAD palmitoylation inhibitor, MGH-CP1, combined with its analogs, inhibited the stem-like properties of cancer cells, prevented excessive tumor growth, and suppressed tumorigenesis both in vitro and in vivo [205]. The sensitivity to MGH-CP1 is significantly correlated with YAP dependence in various tumor cell lines, including NSCLC. These studies indicated that TEAD palmitoylation inhibitors combined with inhibitors of other oncogenic factors can synergistically kill tumors in NSCLC, suggesting the potential role of TEAD inhibitors in treating drug-resistant NSCLC.

### Potential biomarkers that may benefit from drugs targeting the Hippo pathway

How to screen patients who can potentially benefit from drugs targeting the Hippo pathway remains to be known. Table 3 shows candidate biomarkers targeting the Hippo pathway. Genetic alterations affecting the Hippo pathway in NSCLC have been identified. For example, pan-cancer analysis of TEADs showed that approximately 5% of cancers exhibit amplification of TEAD copy numbers. For instance, lung squamous cell carcinoma carries TEAD2, TEAD3, and TEAD4 amplification, and lung adenocarcinoma carries TEAD3 amplification [83]. In pancreatic ductal carcinoma and breast cancer, the overexpression of TEAD or nuclear accumulation is related to poor survival [87, 89]. Additional research is required to determine the predictive significance of TEAD in lung cancer. However, in addition to TEAD, the expression of YAP/TAZ should also be taken into consideration. YAP and TAZ frequently exhibit amplification or overactivation in various human cancers, including lung cancer [25, 26]. Earlier studies have indicated that the amplification of YAP1 and WWTR1, which encode YAP and TAZ, respectively, has been observed in 16% of lung squamous cell carcinoma [206]. In addition, studies have reported a significant correlation between sensitivity to TEAD palmitoylation inhibitor, MGH-CP1, and YAP dependence in NSCLC [205]. Hence, the amplification of YAP could serve as a potential biomarker for predicting the efficacy of TEAD inhibitors in NSCLC. TAZ-CAMTA1 fusion in epithelioid hemangioendothelioma (EHE) [207, 208], and NF2 deletion in MPM [57] can also predict response to TEAD inhibitors; however, these genetic alterations are extremely rare in lung cancer [57]. In addition, mutations and deletions of SAV1 and LATS1/2 are also associated with the proliferation of various tumors [61, 62, 209], and their predictive role in lung cancer needs further studies. Although genetic alterations in the key components of the Hippo pathway are uncommon in lung cancer, the significant relationship between genetic alterations and the efficacy of targeted therapy for Hippo pathway indicates that techniques for detecting these genetic alterations, such as the next-generation sequencing (NGS) technology, may help identify potential candidates who will obtain the utmost benefit. Furthermore, NGS may reveal additional pathogenic genetic changes.

**Table 3:** Potential Biomarkers targeting the Hippo pathway.

Biomarker	Type	Model	Test method	Result	Reference
NF2	Fusion	451 LUAD, 251 LUSC, 9 LCLC, 11 LACC and 24 NSCLC cell lines	RNAseq	NF2-OSBP2 (LUSC) and NF2-MORC2 (LUSC) fusions led to loss of function of NF2.	[48]
	Mutations	Common solid cancers and acute leukemia, including 45 NSCLC (24 LUAD, 21 LUSC)	SSCP assay	NF2 exon 13 c.1351 G > A was found in one LUSC (1/45; 2.2%).	[52]
LATS1/2	Fusion	451 LUAD, 251 LUSC, 9 LCLC, 11 LACC and 24 NSCLC cell lines	RNAseq	LATS1-LACE1 fusion in LUSC and PCMT1-LATS1 fusion in LUAD) impair their cancer inhibition function.	[49]
	Mutation	51 LUAD, 67 LUSC, 11 LCLC, and 13 lung cancer cell lines	DHPLC detection	Nine different genetic alterations were detected in LATS2, mostly occurring in advanced LUSC.	[46]
	mRNA levels	40 LUAD, 38 LUSC, 6 LCLC and adjacent healthy tissue	qRT-PCR	mRNA expression of LATS2 decreased in all tumors, especially in LCLC.	[46]
YAP	Fusion	451 LUAD, 251 LUSC, 9 LCLC, 11 LACC and 24 NSCLC cell lines	RNAseq	YAP1-BIRC2 fusion in LUSC, and YAP1-ENSG0000254968 fusion in LUAD retained the tumor-promoting function of YAP.	[49]
	mRNA levels	9,125 tumor samples across 33 cancer types from TCGA, including LUAD and LUSC.	TCGA database	The combined amplification frequency of YAP1 and TAZ ranged from 0% to 19%, with LUSC ranking second and LUAD eighth among 33 cancer types.	[26]
	mRNA levels	Four human lung tumor tissues and adjacent normal lung tissues	RT-PCR	mRNA levels of YAP were higher in tumor tissues compared with their adjacent normal tissues.	[56]
	Protein levels	66 LUAD and 102 squamous cell carcinomas	IHC	In adenocarcinoma, the expression of cyclin A was independently correlated with the nuclear expression of YAP. Nuclear expression of YAP was an important predictor of EGFR gene amplification. YAP expression in the cytoplasm was an independent predictor of low pTNM stage (stage I) in LUSC.	[47]
	Protein levels	10 human LUAD tissues	IHC	YAP and downstream targets were associated with a worse prognosis.	[50]
	Protein levels	Four human lung tumor tissues and adjacent normal lung tissues	Immunoblotting, Immunofluorescent staining	Total YAP protein levels and nuclear YAP levels were significantly increased in lung cancer tissue, while cytoplasmic levels of pYAP were higher in adjacent normal lung tissues.	[56]
	mRNA levels	TMA encompassing 196 NSCLC cases	WGS	Higher mRNA levels of TAZ were associated with poor survival.	[48]
TAZ	Fusion	451 LUAD, 251 LUSC, 9 LCLC, 11 LACC and 24 NSCLC cell lines	RNAseq	WWTR1-SLC9A9 fusion in LUAD retained the tumor-promoting function of TAZ.	[49]
	Protein levels	10 human LUAD tissues	IHC	TAZ and downstream targets were associated with a negative prognosis.	[50]
	Protein levels	TMA encompassing 345 NSCLC cases	IHC	Higher protein levels of TAZ were associated with poor survival.	[48]
TEAD	mRNA levels	TCGA database: 32 tumor types, including 515 LUAD and 498 LUSC	TCGA database	TEAD2, TEAD3, and TEAD4 were overexpressed in LUSC; TEAD3 was overexpressed in LUAD.	[83]

*NSCLC* non-small cell lung cancer, *LUAD* lung adenocarcinoma, *LUSC* squamous cell carcinoma, *LCLC* large cell lung cancer, *LACC* large cell lung cancer, *SSCP* single-strand conformation polymorphism assay, *pTNM* pathologic TNM, *TCGA* The Cancer Genome Atlas, *TMA* tissue microarray, *IHC* Immunohistochemistry, *RT-PCR* Reverse Transcription-Polymerase Chain Reaction, *WGS* whole-exome sequencing, *RNAseq* RNA sequencing, *DHPLC* denaturing high-performance liquid chromatography, *NF2* neurofibromin 2, *LATS* large tumor suppressor kinase, *YAP* Yes-associated protein, *TAZ* transcriptional co-activator PDZ-binding motif, *TEAD* transcriptional-enhanced associate domain, *EGFR* epidermal growth factor receptor.

Due to the limited occurrence of genetic alterations affecting different elements of the Hippo pathway in lung cancer, a more practical approach for stratifying tumors could be based on protein expression. Using immunohistochemical (IHC) techniques, Drexler et al. [210]. evaluated the protein levels of different constituents of the Hippo pathway in 103 patients with pancreatic ductal carcinoma. The results showed that the Hippo pathway was activated in non-metastatic lesions, and the expression of MST1, MST2, pLATS, pYAP, and 14-3-3 was significantly upregulated (*P* < 0.01). In the metastatic lesions of the liver, the Hippo pathway was inhibited, and LATS1, LATS2, YAP, TEAD2, and TEAD3 were significantly upregulated (*P* < 0.01), and high pYAP expression was associated with better OS and DFS. Tang et al. [88]. observed that the protein levels of TEAD4 were markedly increased in the colorectal adenoma (CRA) tissues. Furthermore, patients who exhibited elevated TEAD4 expression in the normal colon mucosa had an increased risk of relapse following polypectomy. In addition, the study demonstrated that inhibiting TEAD4 expression effectively suppressed the proliferation of colorectal cancer cells in vitro and inhibited tumor growth in vivo. In mouse models of 4NQO-induced HNSCC, it was observed that higher levels of TEAD4 immunostaining were correlated with the progression of the disease. The protein abundance of TEAD4 in IHC staining exhibited a substantial increase in HNSCC clinical specimens compared to control tissues [90]. The aforementioned studies indicate that the protein expression levels of components within the Hippo pathway could function as a candidate biomarker for identifying suitable patients and predicting the effectiveness of drugs targeting this pathway.

### Future outlook

This review offers a thorough overview of the involvement of the Hippo pathway and its downstream molecules, namely YAP/TAZ and TEAD, in the progression and drug resistance of NSCLC. There are still some urgent issues that need to be addressed. First, the Hippo pathway can interact with various carcinogenic signaling pathways, affecting the development and drug resistance of NSCLC. However, it is currently unclear how the Hippo pathway interacts with different signaling pathways and the precise mechanisms underlying the crosstalk between the Hippo pathway and various signaling pathways in NSCLC to produce a coordinated tumor-promoting effect. Second, due to the rarity of genetic alterations affecting different elements of the Hippo pathway in NSCLC, it is crucial to screen candidate patients who may benefit from drugs targeting this pathway. The protein expression of YAP/TAZ and TEAD may be a new target for future research. Third, the development of drugs that target the Hippo pathway represents a promising approach to treat NSCLC and overcome drug resistance. However, further studies are needed on the timing, mode, and sequence of drug use. Fourth, there are other novel therapeutic strategies except for traditional chemoradiotherapy, immunotherapy, and small-molecule inhibitors. CRISPR/Cas9 is an efficient gene editing tool that can precisely excise target sites in the genome by editing the nucleotide sequence of a single guide RNA (sgRNA), correcting pathogenic mutations, or silencing genes associated with disease onset [211, 212]. Its efficacy has been demonstrated for diagnosing and treating various cancers, including lung cancer [213]. Proteolysis targeting chimera (PROTAC) technology selectively degrades disease-related target proteins, providing significant advantages in overcoming drug resistance due to gene mutations or overexpression. It also has the potential to target proteins that are traditionally considered undruggable. Currently, clinical research is underway for PROTAC molecules targeting a range of diseases, including cancer [214]. Exploring the potential use of targeted protein degradation systems, such as PROTAC and CRISPR systems, in targeting the Hippo pathway is also a future research direction. Fifth, although several studies have explored the involvement of the Hippo pathway in NSCLC, YAP, and TAZ have been found to exhibit suppressive effects in neuroendocrine and hematological tumors [215]. Hence, more data are required to fully understand the clinical effectiveness and potential toxicity of targeted therapy for the Hippo pathway in NSCLC. Sixth, due to the interactions between the Hippo pathway and other carcinogenic pathways, investigating the mechanisms of resistance to drugs targeting the Hippo pathway represents a crucial area for future research.

### Supplementary information

Supplementary Material S1
