# Supplementary material for: Hippo pathway in non-small cell lung cancer: mechanisms, potential targets, and biomarkers
Source: Cancer Gene Ther. 2024 Mar 18;31(5):652–66. doi: 10.1038/s41417-024-00761-z (PMC11101353; doi:10.1038/s41417-024-00761-z)
Supplement: Supplementary file 1 — Supplementary Material S1 [file 41417_2024_761_MOESM1_ESM.docx]

**Keywords used in the search**

Hippo or "Hippo pathway" or "Hippo signaling pathway" or YAP or TAZ or TEAD or YAP1 or WWTR1 or "yes-associated protein" or "transcriptional co-activator PDZ-binding motif" or " transcriptional-enhanced associate domain" or MST or LATS or NF2 or "STe20-like kinases

" or "large tumor suppressor kinase" or "neurofibromin 2" (Topic) and "lung cancer" or "non-small cell lung cancer" or NSCLC or "lung adenocarcinoma" or "lung squamous carcinoma" or LUAD or LUSC (Topic)
